# Bibliography

**Published:** 1896-07

**Authors:** 


### Bibliography.

THE PRINCIPLES AND PRACTICE OF DENTISTRY, INCLUDING ANATOMY, PHYSIOLOGY, PATHOLOGY, THERAPEUTICS, DENTAL SURGERY, AND MECHANISM. By Chapin A. Harris, M.D., D.D.S., late President of the Baltimore Medical College, etc. Thirteenth edition. Revised and edited by Ferdinand J. S. Gorgas, A.M., M.D., D.D.S. With twelve hundred and fifty illustrations. Philadelphia: P. Blakiston, Son & Co., 1896.

The publication of the thirteenth edition of the Principles and Practice of Dentistry, the present year, is a sufficient indication of a continuous demand for it as a text-book. When this large volume is compared with the original of Dr. Harris's work the difference as to size and treatment of subjects is most marked. The changes in this book very correctly indicate the progress made in dentistry during the past half-century.

While in many respects defective as a text-book, it is not probably more so than the majority of such works prepared by one or two individuals.

While perhaps none of the present generation of dentists would care to see further editions cease to appear, it must be confessed that the operations in dentistry, even with the condensation made necessary here, have reached such proportions that no one man is capable of properly handling them. It is, therefore, presumed that the text-book of the future will be confined to special subjects, and this, in the opinion of the writer, is the only proper method. All extended works, embracing everything in dentistry, even when written in encyclopædic form, are simply books of reference, and take their places, as such, in every well-arranged dental library, both public and private, but can never become text-books in colleges.

This edition has been quite thoroughly revised by Dr. Gorgas, a most difficult task in view of all the obsolete matter contained in the older editions, and not thoroughly eliminated in any of those published.

The portion devoted to "Dental Pathology and Therapeutics" is, upon the whole, well arranged. Criticism upon it must lie in the direction that it is too brief and not always satisfactory in its line of treatment. The following is quoted as an illustration. "An excellent application is composed of equal parts of the official tincture of iodine and tincture of aconite root, applied to the gum two or three times daily." Not a word of caution as to the use of the tincture of aconite root or as to the *rationale* of the operation. It is simply the old formula. A word of warning as to the possible toxic effects of this agent would not have been out of place in a work supposed to be for the use of students. The description of its application is not only inexact, but the use of aconite for the purpose mentioned, pericementitis, is to be condemned, for it certainly is a paralyzer of tissue, an effect to be by all means avoided in counter-irritation.

In the chapter on "Sensitive Dentine" the author still regards chloride of zinc as not being capable of producing "any deleterious effect on the pulp of the tooth," and this in the face of all the experimental knowledge obtained in the past two years on the effect of coagulants upon this organ.

Cocaine is not mentioned for this purpose, notwithstanding its known effect in reducing a hyperæsthetic condition in dentine more fully since the introduction of electrical osmosis, which was in its infancy when this chapter was, without doubt, prepared.

It is unfortunately the weakness of all books, of a practical character, that before the ink is dry on the pages new methods have been demonstrated and the reader is annoyed by omissions.

The use of bicarbonate of soda might have been stated, with advantage to all practising dentists, for the reduction of sensitiveness in dentine, for certainly no one having used this in the form of a paste—bicarbonate of soda with water—would neglect it as an aid in reducing this pathological condition.

The title of "Necrosis of Teeth," applied to the chapter devoted to partial death of teeth, is a misnomer, and should not have appeared in this connection. The methods of bleaching recommended are about as in the last edition, and equally imperfect and valueless for practical purposes.

It is impossible to extend this review further, nor is it necessary, as the work is too well known to require enlarged notice.

